# Altered Profile of Fecal Microbiota in Newly Diagnosed Systemic Lupus Erythematosus Egyptian Patients

**DOI:** 10.1155/2021/9934533

**Published:** 2021-06-24

**Authors:** Marian A. Gerges, Noura E. Esmaeel, Wafaa K. Makram, Doaa M. Sharaf, Manar G. Gebriel

**Affiliations:** ^1^Medical Microbiology and Immunology Department, Zagazig University, Zagazig, Egypt; ^2^Rheumatology and Rehabilitation Department, Zagazig University, Zagazig, Egypt

## Abstract

**Background:**

Dysbiosis of gut microbiota could promote autoimmune disorders including systemic lupus erythematosus (SLE). Clarifying this point would be of great importance in understanding the pathogenesis and hence the development of new strategies for SLE treatment.

**Aim:**

This study aimed to determine the fecal microbiota profile in newly diagnosed SLE patients compared to healthy subjects and to investigate the correlation of this profile with disease activity.

**Methods:**

Newly diagnosed SLE patients who fulfilled at least four of the American College of Rheumatology (ACR) criteria were enrolled during the study period. Patients with lupus were matched to healthy subjects. SLE activity was evaluated using the Systemic Lupus Disease Activity Index (SLEDAI-2K). Fresh fecal samples were collected from each subject. Genomic DNA was extracted from fecal samples. Quantitative real-time PCR was applied for quantitation of Firmicutes phylum, Bacteroidetes phylum, and *Lactobacillus* genus in comparison to the total fecal microbiota. Results of patients' samples were compared to those of healthy subjects and were correlated to patients' SLEDAI-2K score.

**Results:**

Twenty SLE patients' samples were compared with 20 control samples. There was a significant alteration in SLE patients' gut microbiota. A significantly lower (*p* ≤ 0.001) Firmicutes/Bacteroidetes (F/B) ratio in SLE patients (mean ratio: 0.66%) compared to healthy subjects (mean ratio: 1.79%) was found. *Lactobacillus* showed a significant decrease in SLE patients (*p*=0.006) in comparison to healthy controls. An inverse significant correlation between SLEDAI-2K scores for disease activity and F/B ratio (*r* = −0.451; *p*=0.04) was found. However, an inverse nonsignificant correlation between SLEDAI-2K scores for disease activity and *Lactobacillus* (*r* = −0.155; *p*=0.51) was detected.

**Conclusion:**

Compared to healthy controls, recently diagnosed SLE Egyptian patients have an altered fecal microbiota profile with significant lowering of both F/B ratio and *Lactobacillus* abundance, which is weakly correlated with disease activity.

## 1. Introduction

Gut microbiota is a term used to describe the collection of bacteria, archaea, and eukaryotes colonizing the gastrointestinal tract [1]. The two dominant phyla in the gut microbiota are Firmicutes and Bacteroidetes, representing 90% of the gut microbiota. Actinobacteria, Proteobacteria, Synergistetes, Verrucomicrobia, and Fusobacteria phyla are also present but to a lesser extent [2].

The Firmicutes phylum is composed of more than 200 different genera, such as *Lactobacillus*, *Bacillus*, *Enterococcus*, *Ruminococcus*, and *Clostridium* which alone represents 95% of the Firmicutes phylum. However, the Bacteroidetes phylum consists of two predominant genera, *Bacteroides* and *Prevotella* [3].

In humans, the establishment of gut homeostasis needs a complex interaction between the host immune system and the commensal microbiota. However, when this symbiotic relationship is compromised with alteration of gut microbial function and diversity, a process called dysbiosis, autoimmune diseases may be triggered [4].

The role of gut microbiota dysbiosis in the establishment of different autoimmune diseases, such as rheumatoid arthritis, type 1 diabetes, and inflammatory bowel disease, has been reported [5]. However, the role of intestinal microbiota in systemic lupus erythematosus (SLE) has not been well investigated [6].

Systemic lupus erythematosus is a prototypical autoimmune disorder that damages many organs, including the skin, kidneys, lungs, joints, heart, and brain [4]. Its prevalence ranges from 2 to 5 cases per 10,000 inhabitants [5]. It is remarked by the presence of hyperactive immune cells and aberrant antibodies that bind mainly with nuclear and cytoplasmic antigens [4].

Treatment strategies for SLE patients depend mainly on nonselective immunosuppressant drugs. Unfortunately, not all patients show a good response to such treatment. Besides, these drugs have side effects of major concern [7]. A better understanding of SLE pathogenesis can pave the way for the development of new, more effective, and less hazardous treatment strategies [6].

Several environmental factors have been found to induce the development of SLE, such as diet, modern medicine, and environmental microbes. All these factors can affect the composition of the host gut microbiota; therefore, the study of the dynamics of fecal microbiota in the pathogenesis of SLE is urgently needed [7].

This study aimed to determine the fecal microbiota profile in newly diagnosed SLE patients compared to healthy subjects and to further investigate the correlation of this profile to disease activity.

## 2. Materials and Methods

A case-control study was conducted over one year (January 2020–November 2020) in Medical Microbiology and Immunology Department, Faculty of Medicine, Zagazig University, and Rheumatology and Rehabilitation Department, Faculty of Medicine, Zagazig University Hospitals, Egypt.

This study was approved by the institutional review board (IRB)—Faculty of Medicine, Zagazig University. Written informed consent was obtained from all participants before enrolling in the study. Ethical principles of the Declaration of Helsinki were followed during the preparation of this study.

### 2.1. Participants

Newly diagnosed, treatment-naive SLE cases who fulfilled the inclusion criteria and attended the rheumatology and rehabilitation outpatient clinic, Zagazig University Hospitals, within the study period were included in this study. Patients with lupus were matched to healthy control subjects based on age and sex.

SLE patients with other autoimmune diseases, diabetes mellitus, gastrointestinal diseases affecting the intestinal flora (e.g., irritable bowel syndrome and inflammatory bowel disease), chronic liver disease, or malignancies were excluded from this study. Patients taking medications (such as antibiotics, corticosteroids, or nonsteroidal anti-inflammatory drugs (NSAIDs) and immunosuppressive therapy) and patients with a recent diagnosis of bacterial or parasitic gut infections were also excluded.

Enrolled SLE patients fulfilled at least four of the revised American College of Rheumatology (ACR) criteria for SLE diagnosis [8]. SLE patients were undergone a detailed history and clinical examination. The following routine laboratory assessments were performed for enrolled SLE patients: complete blood picture, erythrocyte sedimentation rate, C-reactive protein, complete urine analysis, 24 h proteinuria, liver and kidney function tests, serum complement 3 level (C3, normal value: 0.9–1.8 g/liter), serum complement 4 level (C4, normal value: 0.1–0.4 g/liter), antinuclear antibodies (ANA) (positive titer > 1/80), and anti-double-stranded DNA antibody (anti-dsDNA) (positive value > 25 IU/ml).

SLE disease activity was evaluated using the Systemic Lupus Disease Activity Index (SLEDAI-2K), which is a revised version of the original SLEDAI [9]. The SLEDAI-2K is a physician-administered instrument assessing 16 clinical features and eight laboratory features in the preceding 10 days. Each item is weighted to give a total score ranging from 0 to 15 points. Patients were classified based on SLEDAI-2K scores into 3 categories: mild activity (SLEDAI: 1–5), moderate activity (SLEDAI: 6–10), and high activity (SLEDAI ≥ 11) [10].

### 2.2. Sample Collection

Fresh fecal samples from different parts of the stool (between 10 and 50 gm per person) were collected from both SLE patients and healthy subjects. The samples were stored in clean containers at −80°C. These samples were used to estimate the quantity of Firmicutes, Bacteroidetes, and *Lactobacillus* in comparison to the total microbiota by real-time PCR.

### 2.3. DNA Extraction

Genomic DNA was extracted from fecal samples using a DNA extraction kit (QIAamp® Fast DNA Stool Mini Kit, Germany) following the manufacturer's instructions.

### 2.4. Real-Time PCR

Quantitative real-time PCR technique was applied for quantitation of Firmicutes, Bacteroidetes, and *Lactobacillus* in comparison to total microbiota using Thermo Scientific Maxima SYBR Green qPCR Master Mix (USA) and Applied Biosystems real-time PCR (StepOne^TM^ Real-Time PCR System, Applied Biosystems Inc., USA).

Standard curves were constructed using twofold serial dilutions of DNA extracted from a pooled sample (pooled from different stool samples of healthy individuals). Standard curves were constructed for bacterial 16S rRNA genes (as a universal gene) to assess the total microbiota and for genes specific to each of Firmicutes, Bacteroidetes, and *Lactobacillus* to assess their quantities.

The primers used were supplied from Thermo Fisher Scientific, USA, and their sequences are listed in Table 1. The singleplex PCR amplifications were performed in a final reaction volume of 20 *µ*l containing 10 *µ*l of 2x SYBR mix (Thermo Fisher Scientific Inc., Maxima SYBR Green qPCR Master Mix (2x), USA), 1 *µ*l (20 pmol) of each primer, and 8 *µ*l of extracted DNA (diluted 1 : 10). Four negative control samples (without DNA templates) were performed for each primer. The amplification protocol consisted of an initial DNA denaturation step at 95°C for 10 minutes, followed by 10 cycles of denaturation at 94°C for 30 seconds, annealing at 60°C for 55 seconds, and extension at 72°C for 45 seconds, then 30 cycles of denaturation at 94°C for 30 seconds, annealing at 50°C for 30 seconds, and elongation at 72°C for 45 seconds. The procedure was completed with a final elongation step at 72°C for 10 minutes. Each reaction was performed in duplicate and repeated twice.

Melting curve analysis was performed after amplification by increasing the temperature from the annealing temperature to 95°C with an increase of 0.5°C per 10 s to monitor the target PCR product specificity and fidelity. The threshold cycle (Ct) values were determined by automatic analysis setting, and mean Ct was calculated. Obtained Ct was plotted against the standard curves, and the bacterial number of each phylum/genus was expressed as a ratio in comparison to the total intestinal microbiota (determined by universal primers) for each sample.

### 2.5. Statistical Analysis

The collected data were statistically analyzed using SPSS software (Statistical Package for the Social Sciences software, version 25). The normality of the distribution of quantitative variables was assessed by the Shapiro–Wilk test. Quantitative normally distributed data were represented as the mean value ± standard deviation (SD) and nonnormally distributed data as median and range. The independent sample *t*-test was used to compare two groups concerning quantitative normally distributed data. Mann–Whitney test was used to compare the two groups concerning quantitative nonparametric data. Spearman and Pearson's correlation coefficients were used to assess the strength and direction of a linear relationship between two variables. Results were considered statistically significant when *p* (probability) values were equal to or less than 0.05.

## 3. Results

A total of twenty newly diagnosed SLE patients (female/male = 18/2) with a mean age of 25.6 ± 6.3 years at the time of diagnosis, as well as twenty healthy subjects (female/male = 16/4) with a mean age of 29.9 ± 6.6 years, were enrolled in this case/control study. Enrolled SLE patients fulfilled at least four of the revised American College of Rheumatology (ACR) criteria for SLE diagnosis. The demographic and clinical data of SLE patients and healthy controls are demonstrated in Table 2.

The mean disease duration was 3.5 ± 1.63 months. SLE disease activity was evaluated using the modified SLEDAI-2K. The mean index score was 9.25 ± 3.9. According to this score, 20% of the studied patients had mild activity, 45% had moderate activity, while 35% had highly active disease (Table 2).

**Figure 1:**
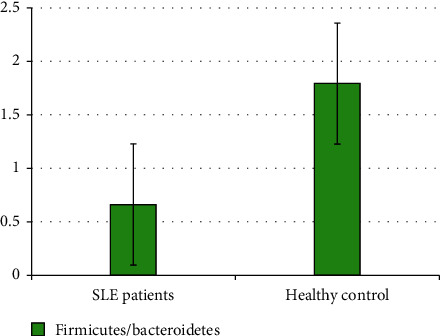
Difference in F/B ratio between SLE patients and healthy controls.

**Figure 2:**
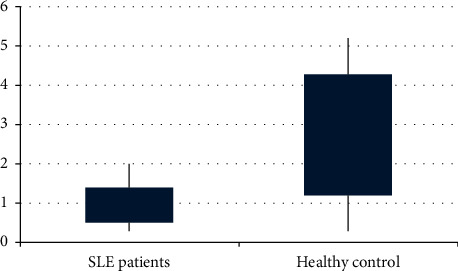
Box-plot analysis for *Lactobacillus* abundance among SLE patients and healthy controls.

The quantity of each phylum/genus of target microbiota was expressed as a ratio in comparison to the total intestinal microbiota for each sample. The mean ± SD (or median, range) ratio of the target fecal microbiota in both cases and controls is shown in Table 3. A significant difference in the ratio of the target microbiota between SLE cases and healthy subjects is demonstrated. The phylum Firmicutes is significantly lowered in SLE patients (28.1% vs. 50.1%; *p* ≤ 0.001), while the phylum Bacteroidetes is significantly enriched in SLE patients (42.96% vs. 29.97%; *p* ≤ 0.001). In turn, the Firmicutes/Bacteroidetes (F/B) ratio is significantly lowered in comparison to healthy subjects (0.66% vs. 1.79%; *p* ≤ 0.001), with an almost 2.7-fold decrease ratio. The genus *Lactobacillus* is significantly lowered in SLE patients compared to healthy controls (0.95% vs. 1.7%; *p*=0.006) (Figures 1 and 2).

**Figure 3:**
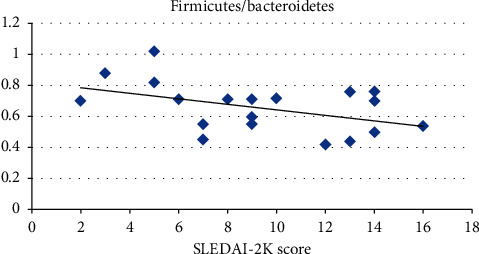
Negative correlation between SLE disease activity and F/B ratio.

**Figure 4:**
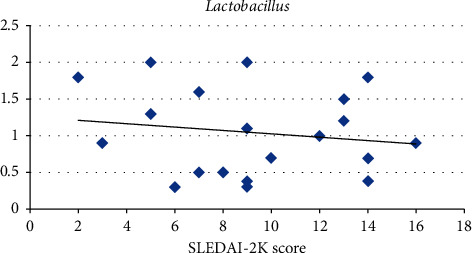
Negative correlation between SLE disease activity and *Lactobacillus* abundance.

An inverse significant correlation between SLEDAI-2K scores for disease activity and F/B ratio (*r* = −0.451; *p*=0.04) has been detected. However, an inverse but nonsignificant correlation between SLEDAI-2K scores for disease activity and *Lactobacillus* abundance (*r* = −0.155; *p*=0.51) has been found (Table 4 and Figures 3 and 4).

## 4. Discussion

This study aimed to determine the profile of fecal microbiota in newly diagnosed SLE patients compared to healthy subjects and to assess the correlation of this profile to disease activity. These insights could have a potential role in the pathogenesis and hence the treatment of SLE, a disease that has an overall estimated prevalence of 6.1/100,000 among adults in Egypt [13]. To the best of our knowledge, this is the first study in our country to assess SLE-associated fecal microbiota dysbiosis among Egyptian SLE patients.

Twenty Egyptian SLE patients (female/male = 18/2) with a mean age of 25.6 ± 6.3 years at the time of diagnosis, as well as twenty healthy subjects (female/male = 16/4) with a mean age of 29.9 ± 6.6 years, were enrolled in this case/control study. The relatively limited sample size recruited in this study is attributed to the relatively low prevalence of SLE in our country [13].

In this study, the phylum Firmicutes was significantly lowered in SLE patients (28.1% vs. 50.1%; *p* ≤ 0.001). The phylum Bacteroidetes was, however, significantly enriched in SLE patients (42.96% vs. 29.97%; *p* ≤ 0.001). The F/B ratio was in turn significantly lowered in comparison to healthy subjects (0.66% vs. 1.79%; *p* ≤ 0.001), with an almost 2.7-fold decrease ratio. Moreover, the genus *Lactobacillus* was significantly lowered in SLE patients compared to healthy controls (0.95% vs. 1.7%; *p*=0.006).

This result comes in agreement with Hevia et al. [5], who studied the gut dysbiosis associated with SLE patients in Spain and reported a significantly lower F/B ratio in SLE patients (median ratio: 1.97) than in healthy subjects (median ratio: 4.86; *p* < 0.002).

Several other studies on SLE patients have found that the abundance of Firmicutes is decreased, and that of Bacteroidetes is increased, demonstrating the decreased F/B ratio in comparison to healthy controls [14–16]. Furthermore, the F/B ratio was consistently reduced in SLE cases, regardless of ethnicity [17]. Furthermore, Katz-Agranov and Zandman-Goddard [18] reported that the F/B ratio was significantly lower in SLE patients even in remission.

Most of these studies were performed in SLE female patients; results within Caucasians and Asians were consistent. However, Luo et al. [7] found no significant difference in the F/B ratio between SLE individuals (both male and female) and controls.

The mechanisms by which intestinal dysbiosis triggers the autoimmunity of SLE are yet to be clarified. Kim et al. [17] stated that intestinal microbiota composition affects intestinal integrity. Translocation of the microbiota because of intestinal epithelial barrier impairment enables microbes to interact with the host immune system, inducing autoantibody production. This is further enhanced in SLE patients by the possible gut colonization with commensals that encode orthologs to the human autoantigen, e.g., Ro60, initiating autoantibody production, as recorded by Greiling et al. [15].

Dysbiosis of F/B ratio in the human gut has been described in association with other autoimmune disorders. Larsen et al. [19] reported that the F/B ratio is decreased in human type 2 diabetes compared to controls. Man et al. [20] reported a decrease in Firmicutes and an increase in Bacteroidetes in association with Crohn's disease. However, an increased F/B ratio has been observed in obesity [21]. In belief, this equilibrium can be modified by long-term shifts in the dietary pattern [22].

In the current study, the *Lactobacillus* genus was significantly lowered in SLE patients compared to controls (0.95% vs. 1.7%; *p*=0.006). This is in agreement with Mu et al. [6] who found marked depletion of *Lactobacillus* in the intestinal microbiota in comparison to controls and added that increasing intestinal colonization by *Lactobacillus* restored the intestinal mucosal barrier function and reduced kidney pathology in a mouse model. However, Zegarra-Ruiz et al. [23] found that *Lactobacillus* was enriched in SLE patients.

Few differences at the genus level were, however, detected by He et al. [14] who mentioned that alterations of the fecal microbiota in SLE patients from China and Spain were shown to be consistent at the phylum level. They rather attributed the differences at the genus level to the differences in the host's genetics and diet.

Results concerning *Lactobacillus* abundance in SLE mouse models were controversial. Kim et al. [17] reported that the effects of abundance of *Lactobacillus* differed among various mouse models of lupus. In the first model, *Lactobacillus* was lacking and found to have a preventive role when used as a probiotic supplement, probably due to its anti-inflammatory properties. However, in the other mouse model, *Lactobacillus* had an opposed role. This has been explained by the multiplicity of the species belonging to the genus *Lactobacillus* that may have different opposing roles in lupus pathogenesis.

An inverse significant correlation between SLEDAI-2K scores for disease activity and F/B ratio (*r* = −0.451; *p*=0.04) was detected in the current study. However, an inverse but nonsignificant correlation between SLEDAI-2K scores for disease activity and *Lactobacillus* was found (*r* = −0.155; *p*=0.51).

This comes in agreement with Xu et al. [24], who stated that SLE individuals had lower species richness diversity (lower Firmicutes/Bacteroidetes ratio and lower *Lactobacilli*) with a high SLE disease activity index. Zhang et al. [25] stated that *Lactobacillus* was found to be negatively correlated with lupus activity, suggesting the usage of probiotics containing *Lactobacillus* to decrease the occurrence and/or severity of lupus flare-up.

However, in mouse models, Luo et al. [7] found that increased abundance of a certain group of *Lactobacillus* in the fecal microbiota might correlate with enhanced disease activity.

Other genera of fecal microbiota, e.g., *Streptococcus*, *Campylobacter*, *Veillonella*, and *Bifidobacterium*, were found to be associated with active and remissive SLE patients, respectively, indicating that SLE patients may exhibit characteristic patterns of fecal microbiota that parallel disease activity [26].

We could not ignore that the lack of diet control in both patients and healthy subjects is one of the limitations in this study. However, previous studies showed that differences in diet had no effect on the fecal microbiota profiles at the phylum level which was investigated in the current study. He et al. [14] mentioned that the profiles of fecal microbiota in SLE patients with different ethnics and different diets, e.g., from China and Spain, were shown to be consistent at the phylum level with few differences at the genus level. They rather attributed the differences at the genus level to the differences in the host's genetics and diet.

In general, the results of the current study along with other studies indicate that obvious dysbiosis of the fecal microbiota is a common finding in SLE patients and may correlate with disease activity. This in turn could serve as a helpful biomarker tool to diagnose and/or predict SLE activity.

## 5. Conclusion

This study demonstrated a significant alteration in the fecal microbiota profile in recently diagnosed treatment-naive SLE Egyptian patients with lowering in both Firmicutes/Bacteroidetes ratio and *Lactobacillus* abundance compared to healthy controls which was negatively correlated to disease activity.

## 6. Limitation of the Study

This study is not without limitations. First, the small sample size included may not represent the entire existing intestinal microbiota. Second, only the two main phyla (Firmicutes and Bacteroidetes) of microbiota and *Lactobacillus* genus were investigated. Third, the changes in diet between SLE patients and healthy subjects were not controlled.

## Figures and Tables

**Table 1:** Primer sets used for amplification reactions.

	Primer sequence (5′-3′)	Reference
All bacteria (universal)	F-ACTCCTACGGGAGGCAGCAGT	[11]
	R-GTATTACCGCGGCTGCTGGCAC	

Firmicutes	F-GGAGYATGTGGTTTAATTCGAAGCA	[12]
	R-AGCTGACGACAACCATGCAC	

Bacteroidetes	F-GGARCATGTGGTTTAATTCGATGAT	[12]
	R-AGCTGACGACAACCATGCAG	

*Lactobacillus*	F-GCAGCAGTAGGGAATCTTCCA	[11]
	R-GCATTYCACCGCTACACATG	

Nucleotide symbol: Y = C or T; R = A or G.

**Table 2:** The demographic and clinical data of the study subjects.

		SLE patients (*n* = 20)	Healthy control (*n* = 20)
Demographic data	Age (years), mean ± SD	25.6 ± 6.3	29.9 ± 6.6
	Female/male	18/2	16/4
	BMI (kg/m^2^), mean ± SD	25.57 ± 4.02	23.78 ± 3.74
	Disease duration (months), mean ± SD	3.5 ± 1.63	—

Clinical data, *n* (%)	Malar rash	6 (30%)	—
	Photosensitivity	14 (70%)	—
	Arthritis	12 (60%)	—
	Oral ulcer	11 (55%)	—
	Alopecia	3 (15%)	—
	Raynaud's phenomenon	11 (55%)	—
	Seizures	1 (5%)	—
	Psychosis	3 (15%)	—
	Nephritis	4 (20%)	—
	Fever > 38	6 (30%)	—

Laboratory assessments	ESR (mm/h), mean ± SD	58.75 ± 34.8	9.05 ± 2.1
	CRP (mg/l), median (range)	4.2 (0.5–43)	2 (0.5–4)
	Serum C3 (g/liter), mean ± SD	0.93 ± 0.65	—
	Serum C4 (g/liter), mean ± SD	0.17 ± 0.1	—
	Positive ANA, *n* (%)	20 (100%)	—
	Positive anti-dsDNA, *n* (%)	17 (85%)	—

Liver function tests	ALT (U/L), mean ± SD	24.22 ± 9.9	—
	AST (U/L), median (range)	21.95 (9.8–76.9)	—

Kidney function tests	BUN (mg/dl), median (range)	14.5 (6.3–29)	—
	Creatinine (mg/dl), median (range)	0.65 (0.36–1.42)	—

Disease activity SLEDAI-2K scores	SLEDAI-2K scores, mean ± SD	9.25 ± 3.9	—
	Mild activity (SLEDAI: 1–5), *n* (%)	4 (20%)	—
	Moderate activity (SLEDAI: 6–10), *n* (%)	9 (45%)	—
	High activity (SLEDAI ≥ 11), *n* (%)	7 (35%)	—

ESR: erythrocyte sedimentation rate; CRP: C-reactive protein; C3: complement 3; C4: complement 4; ANA: antinuclear antibodies; anti-dsDNA: anti-double-stranded DNA antibody; ALT: alanine aminotransferase; AST: aspartate aminotransferase; BUN: blood urea nitrogen; SLEDAI: Systemic Lupus Disease Activity Index.

**Table 3:** The mean (or median) ratio of target fecal microbiota in SLE patients and healthy subjects.

Fecal microbiota	SLE patients (*n* = 20)	Healthy control (*n* = 20)	*p* value
Firmicutes (%) (mean ± SD)	28.1 ± 7.3	50.1 ± 9.5	<0.001^*∗*^HS
Bacteroidetes (%) (mean ± SD)	42.96 ± 10.3	29.97 ± 8.6	<0.001^*∗*^HS
Firmicutes/Bacteroidetes ratio (%) (mean ± SD)	0.66 ± 0.16	1.79 ± 0.59	<0.001^*∗*^HS
*Lactobacillus* (%) (median (range))	0.95 (0.3–2.0)	1.7 (0.3–5.2)	0.006 ^*∗∗*^S

^*∗*^ *t*-test of significance.  ^*∗∗*^Mann–Whitney test for nonparametric data. *p* < 0.05 is statistically significant (S); *p* < 0.001 is highly significant (HS).

**Table 4:** Correlation between disease severity and target fecal microbiota in SLE patients.

Fecal microbiota (SLE)	Disease activity (SLEDAI-2K scores)	
	*r*	*p* value
F/B ratio	−0.451	0.04 S
*Lactobacillus*	−0.155	0.51 NS

*r*: Spearman and Pearson's correlation coefficients.

## Data Availability

All the data used to support the findings of this study are included within the article.
